# Supplementary material for: Transcription Factors in the Fungus Aspergillus nidulans: Markers of Genetic Innovation, Network Rewiring and Conflict between Genomics and Transcriptomics
Source: J Fungi (Basel). 2021 Jul 25;7(8):600. doi: 10.3390/jof7080600 (PMC8396895; doi:10.3390/jof7080600)
Supplement: Supplementary file 1 [file jof-07-00600-s001.zip › TableS1_Oligonucleotides_used_2020_12_24.pdf]

Table S1: Oligonucleotides used in this study.

Name	Sequence (5′-3′)
BrlA-PP1	CAGCCGGGTACTGGAAGCACC
BrlA-GSP2	TTCATCCCAGCCGTCCAGGC
BrlA-GFP1	GCCTGGACGGCTGGGATGAAGGAGCTGGTGCAGGCGCTGGAGCC
BrlAGFP2	AGATCAGCCCTCTTTGTTTCTGTTTCAGTCTGAGAGGAGGCACTGATGCG
BrlA-GSP3	TGAAACAGAAACAAAGAGGGCTGATCT
BrlA-GSP4	CCGCTTCCTACCCCGAATGG
BrlA-ZnFDw	CTGCACCTGCTTGATGACCTGTGG
MsnAZnFforBrlAUp	CGATTGCGACCACCCCGTTTGCTGTGAGTCCTGGCGTGCTGGGCAAGG
MsnAZnFforBrlADw	CCACAGGTCATCAAGCAGGTGCAGACATTCGTCTGCAATCTTTGCTCCCGC
BrlA-ZnFUp	AGCAAACGGGGTGGTCGCAATCG
BrlAp2Dw	GAGGAAGTGGTAAACTGGCGGATGG
BrlA±(+1)	GAAGATCTCGCCGCTCCTCCTCC
An10192-PP1	CGACCAAAGAAGCCGCGATCACAGG
An10192-PP2	CATTGGCGTTGTCTGATTCAAGTGTGTCTTCG
An10192-GSP3	TAACTCTTATTGATGCGCCTAGCTGTGTTCTTTGAGC
An10192-GSP4	CGAAGGACTCTACGTAGACCTCGTGGGC
An10192-SMP1	CGAAGACACACTTGAATCAGACAACGCCAATGACCGGTCGCCTCAAACAATGCTCT
An10192-GFP2	GCTCAAAGAACACAGCTAGGCGCATCAATAAGAGTTAGTCTGAGAGGAGGCACTGATGCG
An10192-sPP1	CCACTGCCCCTGGTTCCAAGAAAGC
An10192-sGSP4	GGCCGCTAACACTACCCAATATCACACGC
An1500-PP1	CGTTTCGTATTGGAGGTATGGCAAGCG
An1500-PP2	CATCATTTTGACGGTGTTGGATCGC
An1500-GSP3	GATGGCTTGATTTGCGAGCTGGAATTGG
An1500-GSP4	GCATCTGCAATGACATGTCCCTCACC
An1500-SMP1	GCGATCCAACACCGTCAAAATGATGACCGGTCGCCTCAAACAATGCTCT
An1500-GFP2	CCAATTCCAGCTCGCAAATCAAGCCATCGTCTGAGAGGAGGCACTGATGCG
An1500-sPP1	GTCAAAGCACAATTGGTGATGGCACG
An1500-sGSP4	CAGCGTGATAAAGGAGGCAATTCGAAGC
An2001-PP1	GAAGCTAGTAGTGCAGCGTCAGCCACC
An2001-PP2	CATGGTCACTGTAGCCTATATCCAGAGATAAAGACAAC
An2001-GSP3	GCGAACTAGAACGACTGAATCTAAGCACATATATATGGC
An2001-GSP4	GGATCTTTTGCGGCAGGCACC
An2001-SMP1	GTTGTCTTTATCTCTGGATATAGGCTACAGTGACCATGACCGGTCGCCTCAAACAATGCTCT
An2001-GFP2	GCCATATATATGTGCTTAGATTCAGTCGTTCTAGTTCGCGTCTGAGAGGAGGCACTGATGCG
An2001-sPP1	CTGCGGAAAGGGCACTTACTTGCG
An2001-sGSP4	GGAGGCGAAGCAACTTAGTCAGTCGG
An4324-PP1	CTCGCCGCAATTGAGAGTCACCG
An4324-PP2	CATTGTGGACGGTGGTAAATATGTACGAGAAG
An4324-GSP3	TAACGCATTCTCTCTTTCTTCTCGCCAGATGC
An4324-GSP4	CGAATCCGAATCCGTCTCCGAACCG
An4324-SMP1	CTTCTCGTACATATTTACCACCGTCCACAATGACCGGTCGCCTCAAACAATGCTCT
An4324-GFP2	GCATCTGGCGAGAAGAAAGAGAGAATGCGTTAGTCTGAGAGGAGGCACTGATGCG
An4324-sPP1	GATGCGCGAGGTCATTCGGGACG
An4324-sGSP4	CATCCGTATAAGGCAAGCTGCGTCACG
An4527-PP1	CATTGACTGCTTGAAGGGAGATGTGACC
An4527-PP2	CATATTGGTCAATGTGCAAGCTTTCCAATCACG
An4527-GSP3	TAGATATCGAACGGCTGTGACGACGTAACG
An4527-GSP4	CCCACAGCCACAGATAGACTTCCATTCG
An4527-SMP1	CGTGATTGGAAAGCTTGCACATTGACCAATATGACCGGTCGCCTCAAACAATGCTCT
An4527-GFP2	CGTTACGTCGTCACAGCCGTTCGATATCTAGTCTGAGAGGAGGCACTGATGCG
An4527-sPP1	GTGAGCTTACCAAGTGCCCGACCTGC
An4527-sGSP4	CTGATGACGATAATCTGGGGTAGGAAGGC
