# Supplementary figures and images for: Transcription Factors in the Fungus Aspergillus nidulans: Markers of Genetic Innovation, Network Rewiring and Conflict between Genomics and Transcriptomics
Source: J Fungi (Basel). 2021 Jul 25;7(8):600. doi: 10.3390/jof7080600 (PMC8396895; doi:10.3390/jof7080600)

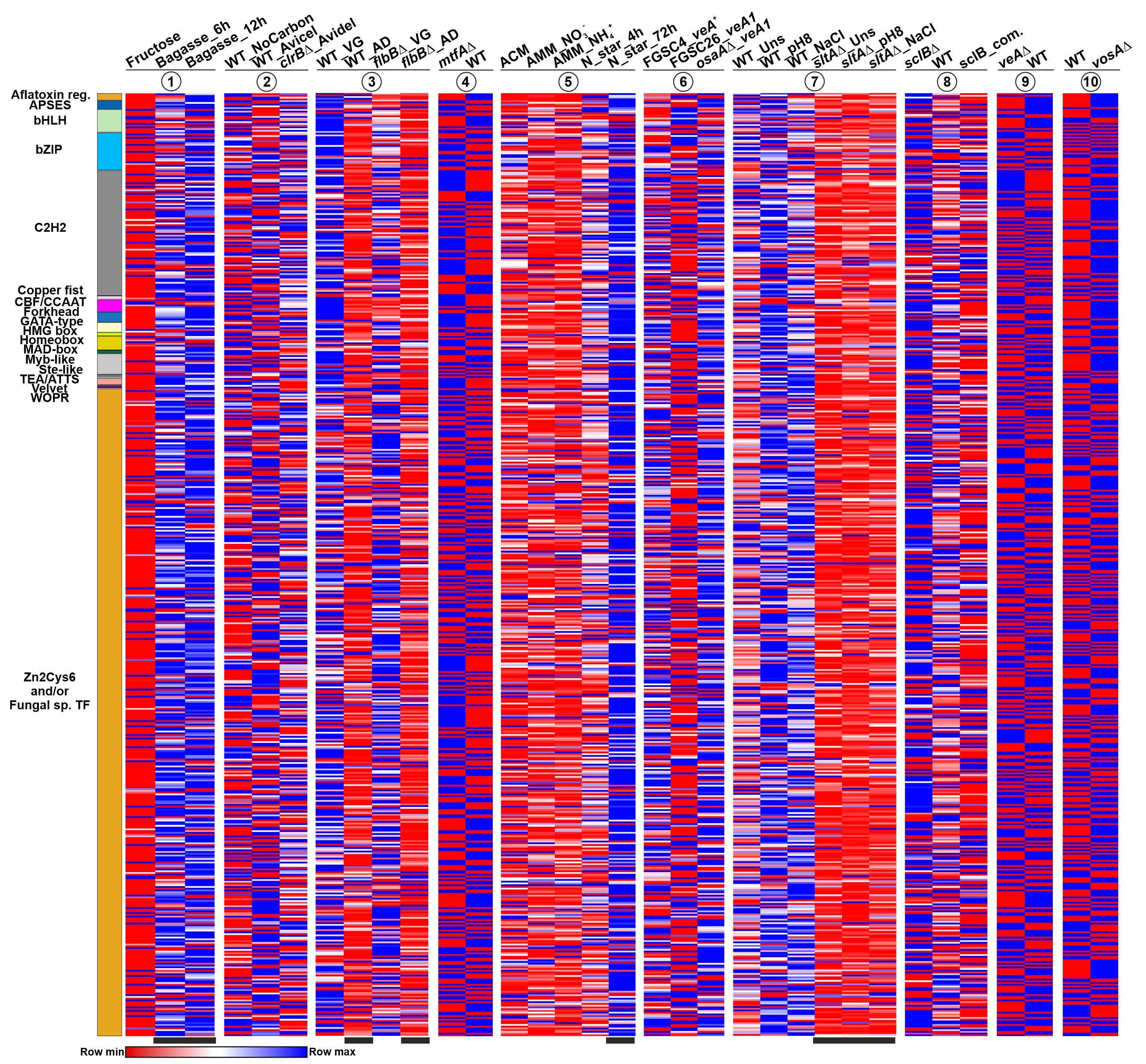

Supplement: Supplementary file 1 [file jof-07-00600-s001.zip › Figure S1_TFs Heatmaps_All_Families_2021_07_15.jpg]

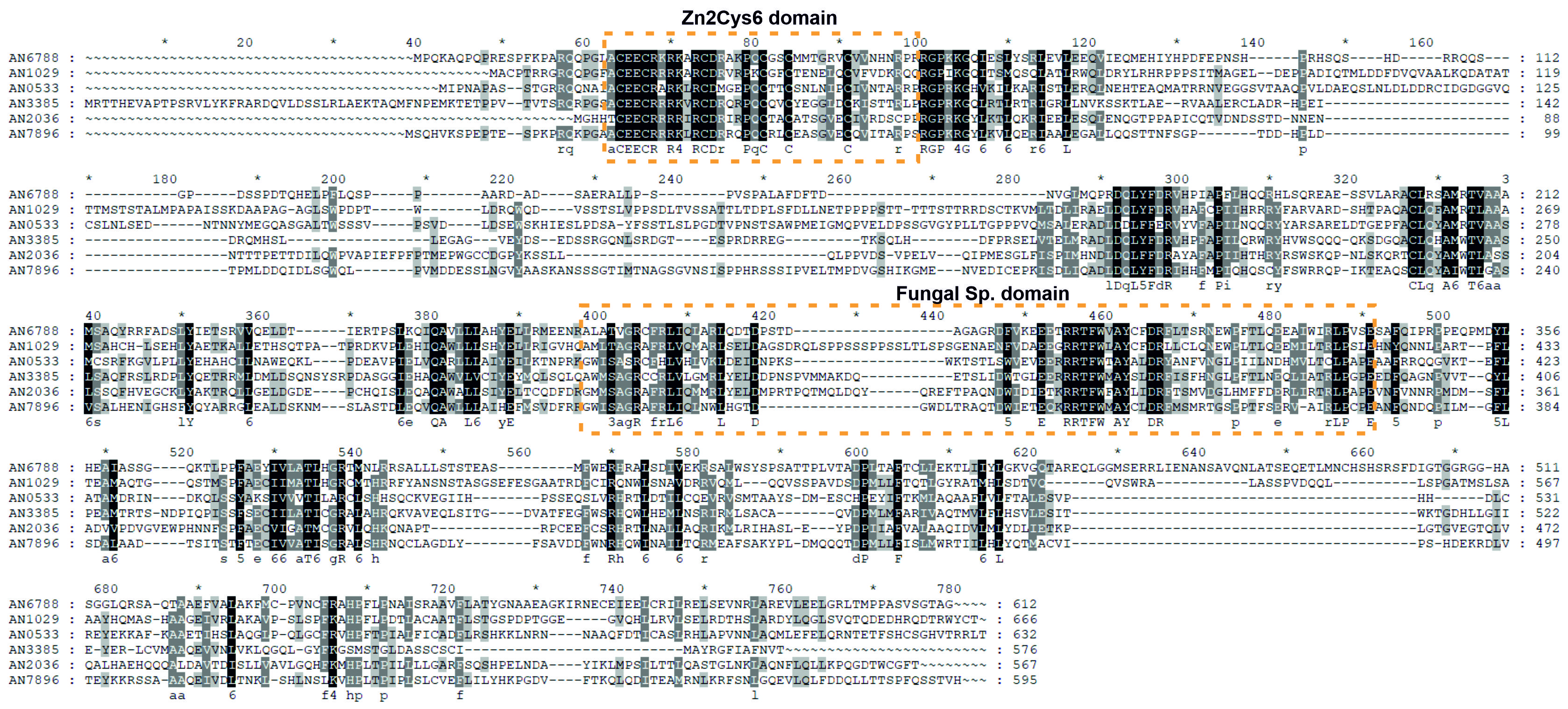

Supplement: Supplementary file 1 [file jof-07-00600-s001.zip › Figure S2_Alignment_An3385_Zn2Cys6_Paralogs_2021_07_15.jpg]

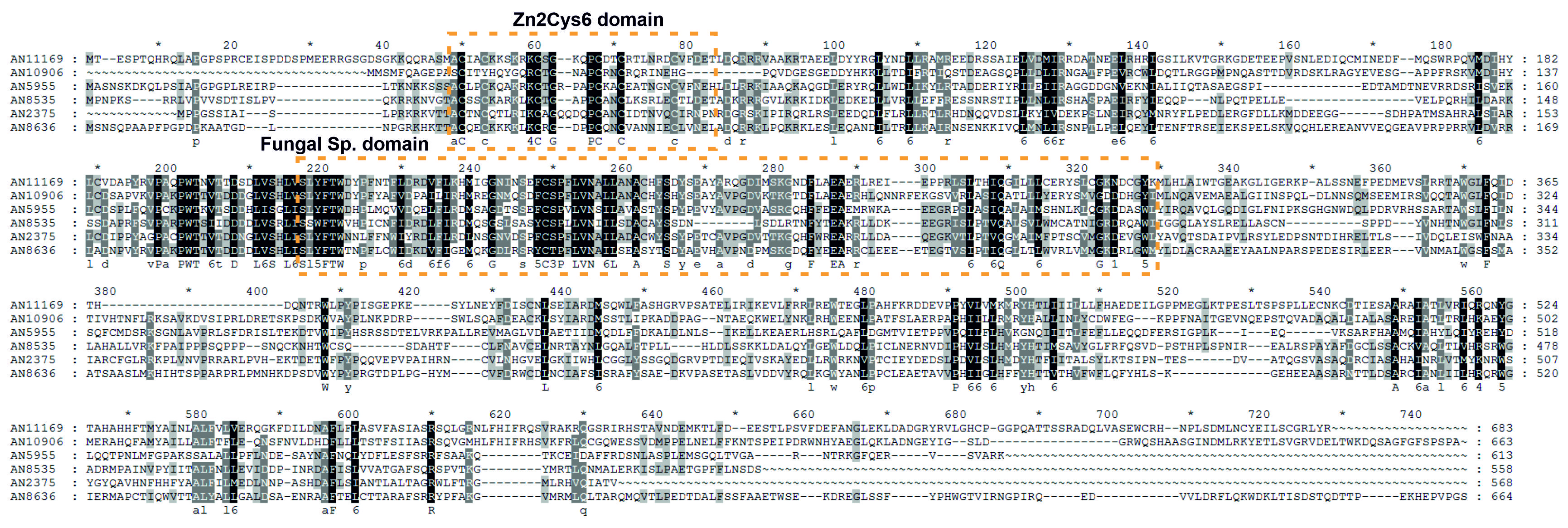

Supplement: Supplementary file 1 [file jof-07-00600-s001.zip › Figure S3_Alignment_An11169_Zn2Cys6_Paralogs_2021_07_15.jpg]

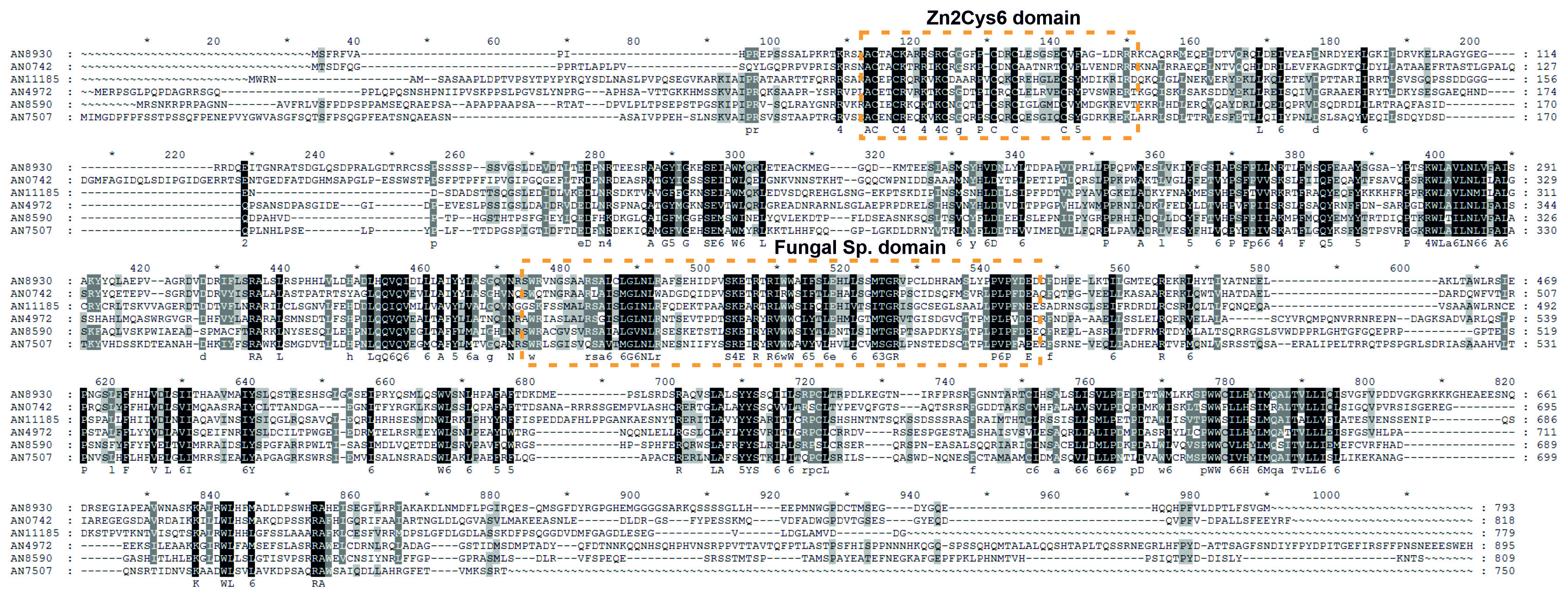

Supplement: Supplementary file 1 [file jof-07-00600-s001.zip › Figure S4_Alignment_An8930_Zn2Cys6_Paralogs_2021_07_15.jpg]

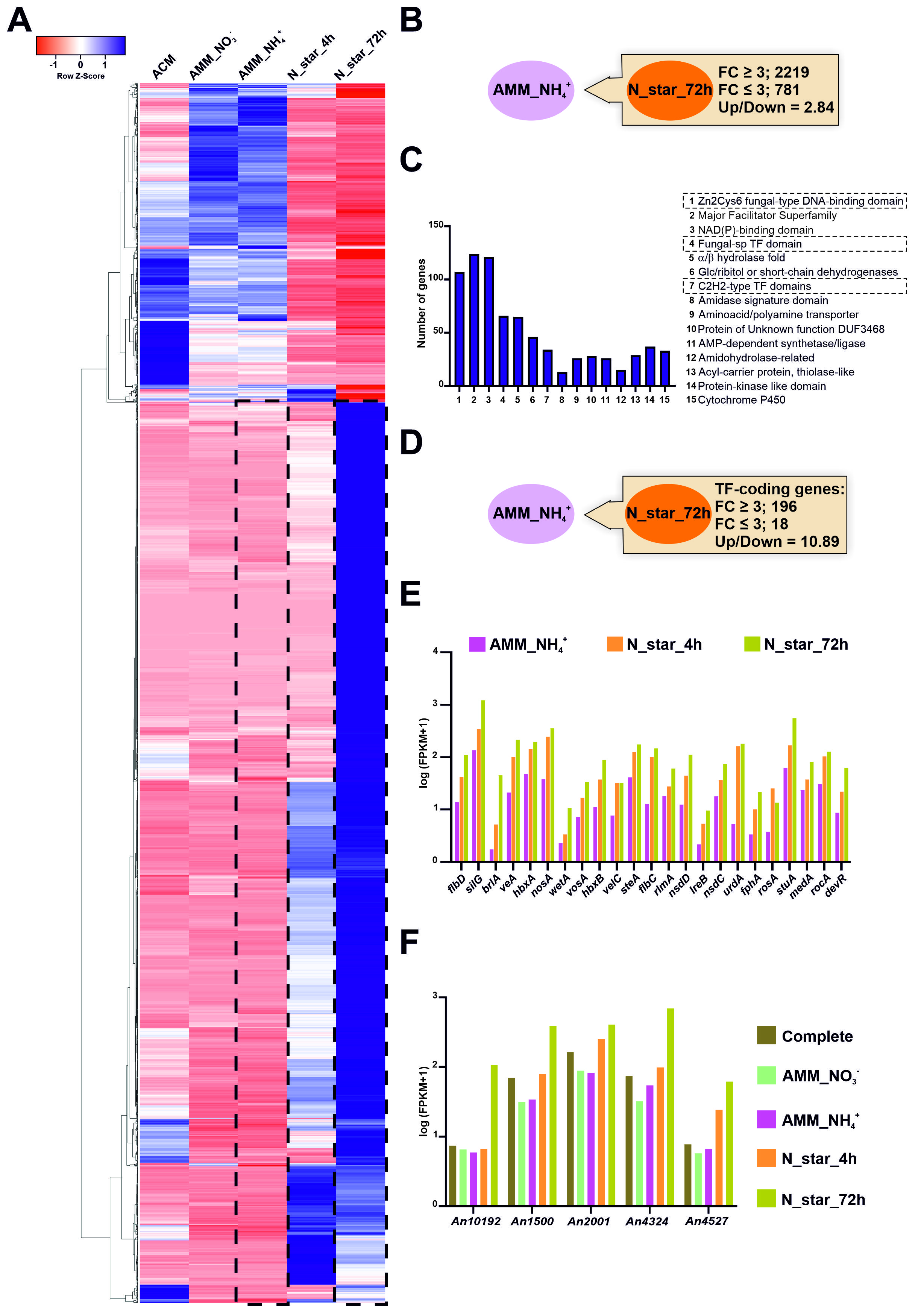

Supplement: Supplementary file 1 [file jof-07-00600-s001.zip › Figure S5_N_starvation_RNAseq_2021_07_15.jpg]

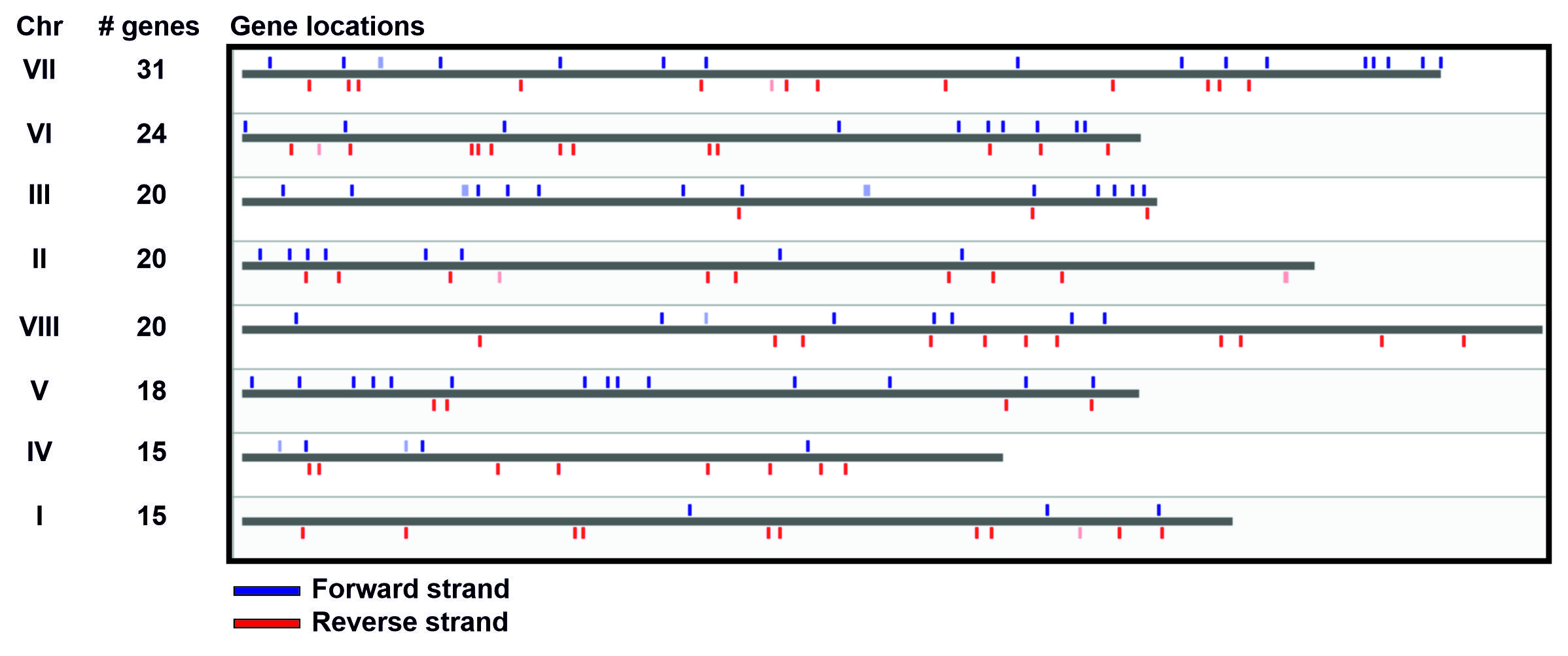

Supplement: Supplementary file 1 [file jof-07-00600-s001.zip › Figure S6_Distribution_Zn2Cys6_CDHit_Anidulans_Genome_2021_07_15.jpg]
